# T-4 Measuring What Matters: Validating the HANDFULS Hand Outcome Tool in Pediatric Burn Survivors

**DOI:** 10.1093/jbcr/irag033.004

**Published:** 2026-04-06

**Authors:** Ingrid Parry, Sandra Taylor, Michelle A James, David G Greenhalgh

**Affiliations:** Shriners Children's - Northern California, Sacramento, CA; University of California Davis, Davis, CA; Shriners Hospitals for Children, Sacramento, CA; Shriners Hospitals for Children, Sacramento, CA

## Abstract

**Introduction:**

Hand and finger burns are common in children and often lead to deformity and functional limitations. Despite the importance of hand function during development, few outcome tools are designed or validated for the pediatric burn population. HANDFULS is a new performance-based measure that evaluates in-hand manipulation and palmar workspace volume (PWV), two frequent impairments after burn injury.

The aims of this study were to use HANDFULS to: 1) establish functional recovery trajectories for children during two years of recovery, and 2) compare hand burn outcomes to the performance of age-matched non-injured peers.

**Methods:**

We conducted a prospective longitudinal cohort of 119 children (165 burned hands), assessing hand function at 2, 6, 12, and 24 months post-burn. Mixed-effects models with random intercepts for hand clustering evaluated change over time while controlling for confounders. PWV and HANDFULS scores were compared with non-injured peers and analyzed by surgical status. Reliability was examined using intraclass correlation coefficients.

**Results:**

Participants averaged 8.5 years of age with mean TBSA 12.7%. Surgery was performed on 41% of hands, most commonly involved split-thickness skin grafting (30.3%). Table 1. PWV improved by 1.5 marbles (*p*=.005) and HANDFULS scores by 1.2 sec/marble (*p*=.002) over two years, with greatest gains observed between 2–6 months. Surgical patients had lower PWV at 2 months(–2.22, *p*=.001) and 6 months (1.28, *p*=.036) but there was no difference in HANDFULS scores, indicating preserved dexterity despite reduced PWV. Compared to non-injured peers, 45.2% (PWV) and 31.8% (HANDFULS) of children with hand burns were within normal limits at 2 months, which increased to 77.6% and 78.1%, respectively by 24 months. Figure.

**Conclusions:**

HANDFULS is a simple, feasible and burn-specific hand outcome measure that demonstrated validity, reliability, and responsiveness. This longitudinal study with a robust sample of pediatric burn survivors demonstrated that the tool can objectively track hand recovery after burns and skin grafting and benchmark outcomes to noninjured peers. Its specificity to burn-specific hand problems allows it to better support clinical decision-making, guide rehabilitation, and improve long-term care after burn injury.

**Applicability of Research to Practice:**

HANDFULS fills a critical gap in pediatric burn assessment and can support tailored interventions.

**Funding for the study:**

Institutional Grant 72 001.

